# Why Do Indians Have Such Good Teeth?

**Published:** 1886-08

**Authors:** 


### "WHY DO INDIANS HAVE SUCH GOOD TEETH?"

The secret of the extraordinary longevity of the Indians is their simple diet and regular habits. They themselves say that when an Indian goes into service and eats the food of the white man, the Indian's teeth begin to decay. Their perpetual grinding on tortillas keeps the teeth white, and the lime in the tortilla makes tooth bone.—*Exchange.*

Such paragraphs as the above are the consequence of pseudo-scientific writing by men who know nothing of the facts of the case. What particular Indians are referred to is not stated, but it is to be presumed that the North American Indians are meant. In that supposition, the heading of this article would remind one of the question of Columbus. Why, when a turbot is placed in a full vessel of water, is there no overflow? Long speculation concerning the reason was indulged in, till it occurred to some one to test the matter, when it was found that the water did overflow.

In the first place, Indians do not have good teeth.

Secondly, they are not at all noted for longevity. On the contrary, all the races of Indians are swiftly dying out, and fast becoming extinct.

Thirdly, Indians are not of regular habits, their only regularity consisting in being regularly irregular.

Fourthly, an Indian never goes into "service," as the English understand the term. He is incapable of submitting to "the regular habits and simple diet" of the white man. He wants little of the whites, except whisky.

Tortillas are corn cakes used in Mexico, and it is rather tough to imagine them as serving for tooth powders.

About the "lime in the tortillas making tooth-bone"—well, that is too funny for anything. Why will professionally scientific journals give currency to such absurdities under the name of scientific teaching? To start out with notoriously false premises, and, based upon them, to weave such a web of sophistry and false deduction, is scarcely worthy a professional journal. It is no excuse to plead ignorance, for such assertions should not be indulged in without the certainty of truth to base them upon.

